# Regional assessment of availability for transcatheter aortic valve implantation in Sweden: a long-term observational study

**DOI:** 10.1093/ehjqcco/qcad076

**Published:** 2023-12-29

**Authors:** Konrad Nilsson, Daniel Lindholm, Jenny Backes, Henrik Bjursten, Henrik Hagström, Johan Lindbäck, Pétur Pétursson, Magnus Settergren, Giovanna Sarno, Stefan James

**Affiliations:** Department of Medical Sciences, Cardiology, Uppsala Universitet, 751 85 Uppsala, Sweden; Department of Medicine, Visby Lasarett, 621 55 Visby, Sweden; Department of Medical Sciences, Epidemiology, Uppsala Universitet, 751 85 Uppsala, Sweden; Department of Medicine, Norrtälje Hospital (TioHundra AB), 761 29 Norrtälje, Sweden; Department of Medical Sciences, Örebro Universitet, 701 82, Örebro, Sweden; Department of Cardiothoracic Surgery, Anaesthesia and Intensive Care, Lund University/Skåne University Hospital, 221 84 Lund, Sweden; Department of Public Health and Clinical Medicine, Umeå University, and Heart Centre, Umeå University Hospital, 901 85 Umeå, Sweden; Uppsala Clinical Research Center, 751 85 Uppsala, Sweden; Department of Cardiology, Sahlgrenska University Hospital, 413 45 Gothenburg, Sweden; Department of Cardiology, Karolinska Institutet, 171 77 Stockholm, Sweden; Department of Medical Sciences, Cardiology, Uppsala Universitet, 751 85 Uppsala, Sweden; Uppsala Clinical Research Center, 751 85 Uppsala, Sweden; Department of Medical Sciences, Cardiology, Uppsala Universitet, 751 85 Uppsala, Sweden; Uppsala Clinical Research Center, 751 85 Uppsala, Sweden

**Keywords:** Transcatheter aortic valve implantation, Aortic stenosis, Equal care, Implementation Health care organisation

## Abstract

**Background:**

Transcatheter aortic valve implantation (TAVI) is an increasingly important treatment option for patients with severe aortic stenosis. Its best implementation is debated, as few centres with high volumes are associated with better outcomes, while centralization might lead to an inferior availability of treatment for patients living far away. The aim of this study was to investigate the implementation of TAVI in Sweden with a focus on regional differences in terms of availability, short-term mortality, and waiting times.

**Methods:**

All patients undergoing TAVI between 2008 and 2020 from the Swedish Transcatheter Cardiac Intervention Registry (SWENTRY) were included. SWENTRY was linked to the National Cause of Death Registry and to publicly available geospatial data from Statistics Sweden.

**Results:**

A total of 7280 patients were included. Over time, TAVI interventions increased markedly, while surgical aortic valve replacement (SAVR) remained constant. There were no statistically significant regional differences in incidence between counties with or without a local TAVI centre (*P* = 0.7) and no clustering tendencies around regions with a local TAVI centre (*P* = 0.99). Thirty-day mortality improved over time without evidence of regional differences. No regional differences in waiting time from decision to intervention were found for TAVI centre regions and non-TAVI centre regions (*P* = 0.7).

**Conclusion:**

This nationwide study indicated no regional differences in terms of availability, short-term mortality, or waiting times. An organization with a few specialized centres was found to be sufficient to provide national coverage of TAVI interventions.

## Graphical Abstract

**Graphical Abstract:**
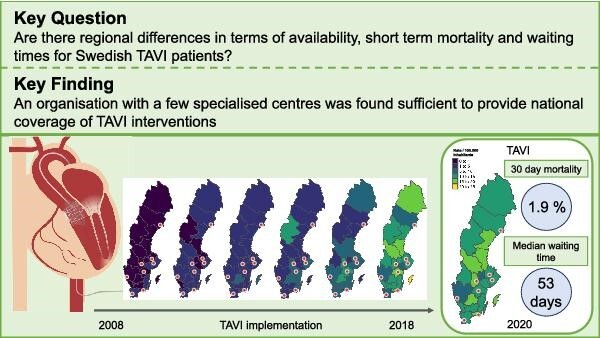

**Abbreviations**
CABG, Coronary artery bypass graft
COPD, Chronic obstructive pulmonary disease
eGFR, estimated glomerular filtration rate
EACTS, European Association of Cardio-Thoracic Surgery
ESC, European Society of Cardiology
ICD-10, International Classification of Diseases—10^th^ Revision
LVEF, Left ventricular ejection fraction
NYHA, New York Heart Association
PCI, Percutaneous coronary intervention
PVD, Peripheral vascular disease
SAVR, Surgical aortic valve replacement
SWEDEHEART, Swedish Web-system for enhancement and development of evidence-based care in heart disease evaluated to recommended therapies
SWENTRY, Swedish Transcatheter Cardiac Intervention Registry
TAVI, Transcatheter aortic valve implantation

Key Learning Points **What is already known:**

- Transcatheter aortic valve implantation (TAVI) is an increasingly important treatment option for patients with aortic stenosis, and it has enabled treatment of patients who were earlier not suitable for intervention.
- Its implementation on a regional level, in terms of treatment availability and prognosis, is sparsely investigated.

**What this study adds:**

- This study illustrates the regional implementation.
- Geographical distance to a TAVI centre does not seem to be associated with availability or prognosis.
- These findings add information on how to organise health care in a context with universal availability to healthcare and few specialized centres.

## Introduction

Aortic stenosis is a progressive disease that mainly affects older patients and is associated with high mortality and morbidity rates and worse quality of life. The only way to alleviate the patient from aortic stenosis is by aortic valve replacement.^1^ Recently, transcatheter aortic valve implementation (TAVI) has become an important treatment option for aortic valve replacement, which has enabled the treatment of patients who were previously not candidates for surgery.^2^ Its usage is rapidly increasing, as is evidence supporting TAVI for patients who were previously candidates for surgical aortic valve replacement (SAVR). It is currently exceeding SAVR and is believed to keep increasing due to aging populations and patient preferences.^3–7^

With the rapid implementation of a novel treatment option, questions arise regarding the best way to organize and give availability to health care. In current guidelines from the European Society of Cardiology (ESC) and the European Association of Cardio-Thoracic Surgery (EACTS), it is stated that ‘aortic valve interventions must be performed in Heart Valve Centres that declare the local expertise and outcomes data, have active interventional surgery and cardiac surgery programmes on site, and a structural collaborative Heart Team approach’ as a level 1C recommendation.^8^

Earlier studies have indicated an association between greater TAVI volumes and improved outcomes. Nonetheless, the appropriate threshold of a sufficient amount of interventions is unknown.^8,9^ In contrast, the concentration of interventions to a few specialized centres might lead to geographical differences in treatment availability, which especially holds true in countries with decentralized health care systems where local authorities and financial frameworks affect which patients and on what indications are referred.^10,11^

The aim of this study was to investigate the implementation of TAVI in Sweden with a focus on regional differences in terms of availability, short-term mortality, and waiting times and to study corresponding trends in SAVR and the change in the total number of interventions due to aortic stenosis over time using Swedish national registries, which contain full coverage of all aortic valve procedures.

## Methods

### Study population

Data were retrieved from the Swedish Transcatheter Cardiac Intervention Registry (SWENTRY) and the Swedish Cardiac Surgery Registry, which are subregisters of the Swedish Web-system for Enhancement and Development of Evidence-based Care in Heart Disease Evaluated according to Recommended Therapies (SWEDEHEART) registry. The registries, which have been presented in earlier publications,^12–14^ hold detailed information on procedures, periprocedural outcomes, comorbidities, angiography, computed tomography, and echocardiography findings of patients admitted to cardiac care or undergoing cardiac interventions, with national complete coverage. Using personal identification numbers, individual data were then linked together with the National Patient Registry and the Cause of Death Registry. Furthermore, for each individual, their place of residence on a municipality level was collected from SWEDEHEART, and the number of procedures was aggregated according to Sweden's 21 counties, divided into three time periods. Ethical permission was granted from the Swedish Ethical Review Authority (Dnr 2017/455).

The aggregated data were merged with Swedish population data and geodata, which are publicly available from Statistics Sweden.^15,16^

All patients who received a TAVI prosthesis or had at least one diagnosis of aortic stenosis (International Classification of Diseases—10^th^ Revision (ICD-10) code I35.0 or I35.2) in the National Patient Registry and underwent isolated SAVR including reinterventions between 2008 and 2020 were included. No exclusion criteria were adopted.

### Definitions and outcomes of interest

All included variables, such as procedure dates, clinical baseline characteristics, and place of residence, were obtained directly from SWEDEHEART. The date of death was collected from the National Population Registry at the Swedish tax agency. Data from Statistics Sweden were used to find the population numbers for each Swedish county to be able to create rates per 100 000 inhabitants. A reference map that illustrates population density by region is available in the supplementary appendix. Coordinates for each TAVI centre were retrieved from OpenStreetMap (www.openstreetmap.org). Statistics Sweden was also used to find coordinates for each county's geographical borders.

We divided the population into three time periods for presentation purposes. During 2008, only 77 patients underwent TAVI.

The study was reported in accordance to The Strengthening the Reporting of Observational Studies in Epidemiology (STROBE) Statement.^17^

### Statistical analyses

Temporal trends were visualized using maps and area graphs. Spatial correlation of intervention rates was analysed using Monte Carlo simulation of the Moran I statistic. The Moran I statistic consists of an analysis of all regions in pairs. If both neighbouring regions are above or below the mean, they contribute with a positive value. If the neighbours are on different sides of the mean, they contribute with a negative value. An overall positive value indicates similarity of neighbouring regions, which could indicate a positive spatial autocorrelation and a non-random distribution of, in this setting, regional incidences of TAVI procedures. In the analysis, the region of Gotland was excluded because it consists of an island with no adjacent regional borders. A Monte Carlo simulation was then used to generate a distribution of theoretical expected values given that, in this case, TAVI rates are completely randomly spatially distributed. Next, to assess whether the result was statistically significant, the observed Moran I statistic was compared with the Monte Carlo distribution of the statistic under the null hypothesis.^18^ A sensitivity analysis was performed where spatial correlation was analysed in three different age strata.

Mortality rates were compared using the log-rank test. Differences between counties with or without a TAVI centre in times from decision to intervention were analysed with the Wilcoxon Mann‒Whitney test. To analyse regional differences in the population, a standardization by age and sex was performed using both a direct and a regression-based method. To perform the direct method, the expected number of cases in a standard population was divided by the standard population size. In the analysis, age and sex were treated as categorical variables where age was divided into 5-year intervals. In the regression-based model, a standardization using a Poisson regression model was performed where age and sex were treated as continuous variables.

Missing frequencies were expected to be low, and thus the data were presented without imputation strategies. However, the data and missing frequencies were examined to identify possible causes of bias.

All analyses were performed using R Statistical Software (v4.1.1; R Core Team 2021). For the spatial analyses, the packages ‘SPDEP’ and ‘tmap’ were used.^19,20^

## Results

A total of 7280 patients undergoing TAVI were included in the study. There were 843, 1962, and 4475 TAVI patients in the time periods of 2008–12, 2013–16, and 2017–20, respectively. For SAVR, the corresponding numbers were 12 026 patients, out of which there were 4743, 3871, and 3412 in the respective time periods.

The baseline characteristics of patients undergoing TAVI are presented in *Table 1.* The prevalence of prior coronary artery bypass graft or percutaneous coronary intervention (PCI), chronic obstructive pulmonary disease, stroke, and peripheral vascular disease decreased over time. On the other hand, the prevalence of existing diabetes and hypertension remained constant or even increased slightly. In addition, more patients were treated in lower New York Heart Association classes and with preserved left ventricular ejection fraction in the later years. Interventional complications and complications during the index hospitalization decreased markedly; interventional complications decreased from 17.8% in the first time period to 6.0% in the last, and in-hospital complications decreased from 27.2% to 7.5%. Baseline characteristics for patients undergoing SAVR are presented in Supplementary *Table S1*.

**Table 1:** Baseline characteristics

	2008–2012	2013–2016	2017–2020
*N*	843	1962	4475
*Pre procedural:*			
Age: mean (SD)	81.68 (7.41)	81.53 (7.57)	80.91 (6.85)
Sex: male n (%)	434 (51.5)	990 (50.5)	2397 (53.6)
Body mass index (kg/m^2^): mean (SD)	25.99 (4.90)	26.62 (5.35)	26.95 (8.16)
Frailty: n (%)	0 (NA)	375 (20.7)	865 (19.4)
Neuromuscular dis.: n (%)	74 (8.8)	284 (14.5)	594 (13.3)
Myocardial infarction: n (%)	41 (4.9)	130 (6.6)	163 (3.6)
Hypertension: n (%)	609 (72.2)	1448 (73.8)	3448 (77.1)
Diabetes: n (%)	181 (21.5)	486 (24.8)	1160 (25.9)
CABG: n (%)	278 (33.0)	471 (24.0)	685 (15.3)
PCI: n (%)	261 (31.0)	519 (26.5)	1176 (26.3)
COPD: n (%)	183 (21.7)	371 (18.9)	776 (17.3)
Stroke: n (%)	129 (15.3)	266 (13.6)	514 (11.5)
PVD: n (%)	206 (24.5)	344 (17.5)	735 (16.4)
Existing pacemaker: n (%)	0 (NA)	127 (9.9)	475 (10.6)
NTproBNP (ng/L): median [IQR]	2485 [1158, 5908]	2291 [944, 5438]	1580 [646, 3740]
eGFR (mL/min/1.73 m^2^) : mean (SD)	55.89 (19.06)	57.19 (19.71)	60.72 (19.83)
*NYHA class: n (%)*			
I-II	48 (5.7)	207 (10.6)	953 (21.3)
III	652 (77.4)	1447 (73.9)	3040 (68.0)
IV	142 (16.9)	303 (15.5)	478 (10.7)
*LVEF (%)*			
≥50	486 (57.9)	1181 (60.2)	3134 (70.3)
40–49	154 (18.4)	325 (16.6)	672 (15.1)
30–39	134 (16.0)	276 (14.1)	408 (9.2)
<30	65 (7.7)	179 (9.1)	245 (5.5)
*ASclass: n (%)*			
High gradient, preserved EF	434 (52.0)	1024 (52.2)	2713 (60.8)
High gradient, low EF	249 (29.8)	486 (24.8)	821 (18.4)
Low gradient, preserved EF	49 (5.9)	156 (8.0)	421 (9.4)
Low gradient, low EF	103 (12.3)	294 (15.0)	504 (11.3)
Max velocity (m/s): mean (SD)	4.45 (0.67)	4.35 (0.65)	4.34 (0.63)
Mean aortic gradient (mm Hg): mean (SD)	50.18 (16.12)	47.35 (15.47)	46.97 (14.54)
*Aortic regurgitation: n (%)*			
Normal	288 (34.7)	748 (38.4)	1792 (40.9)
Mild	439 (53.0)	1000 (51.4)	2158 (49.2)
Moderate	86 (10.4)	169 (8.7)	339 (7.7)
Severe	16 (1.9)	30 (1.5)	94 (2.1)
*Mitral regurgitation: n (%)*			
Normal	220 (26.8)	533 (27.5)	1546 (35.4)
Mild	445 (54.1)	1097 (56.5)	2298 (52.7)
Moderate	139 (16.9)	273 (14.1)	485 (11.1)
Severe	18 (2.2)	37 (1.9)	33 (0.8)
*Post procedural*			
Complication at lab: n (%)	150 (17.8)	265 (13.5)	269 (6.0)
Complication at ward: n (%)	224 (27.2)	430 (22.3)	334 (7.5)
Post max velocity (m/s): mean(SD)	2.10 (0.48)	2.09 (0.59)	2.18 (0.53)
Post mean aortic gradient (mm Hg): mean (SD)	10.29 (4.47)	10.92 (6.09)	10.67 (5.79)

CABG, coronary artery bypass graft; COPD, chronic pulmonary disease; eGFR, estimated glomerular filtration rate according to the CKD-EPI formula; LVEF, left ventricular ejection fraction; NYHA class, New York Heart Association class; PVD, peripheral vascular disease.

Temporal data and short-term mortality had complete coverage. For baseline characteristics, the variables frailty and new onset pacemaker were not implemented in the registry until 2013 and 2014, respectively, and are consequently not present in the first-time period. In the remaining years, the variables had missing frequencies of 1.3% and 4.9%, respectively. The post mean and max gradients had a missing frequency of approximately 40%. The remaining variables had missing frequencies below 2.5%.

**Figure 1:**
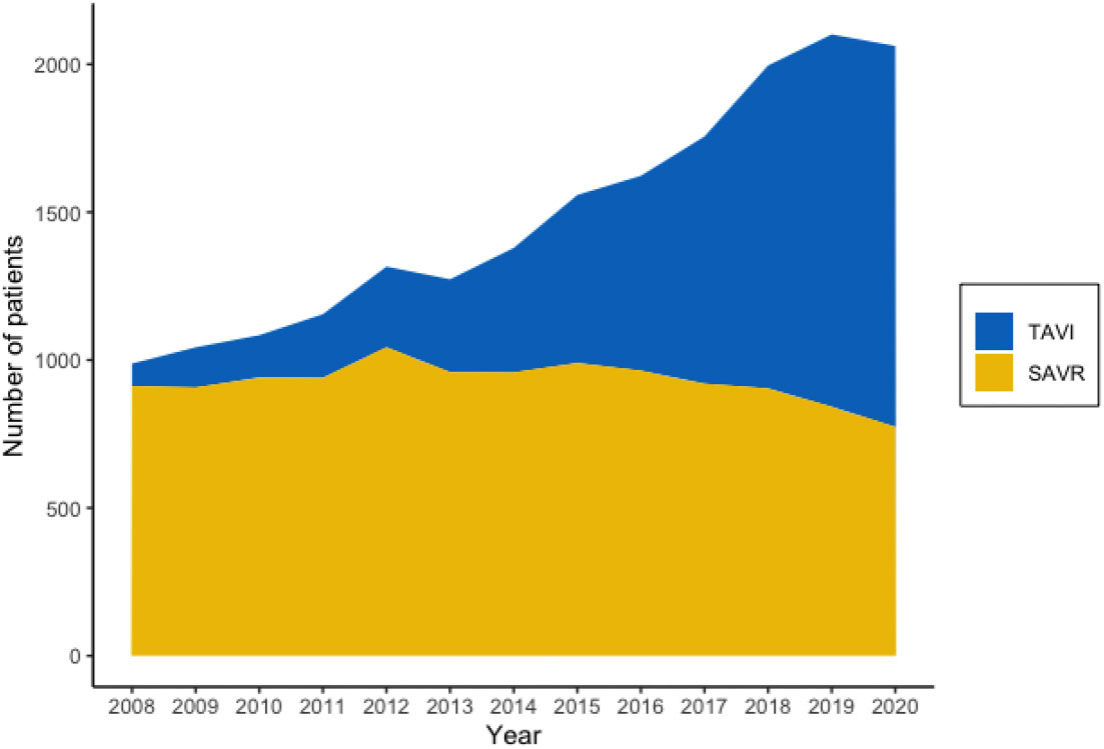
Temporal trends for TAVI and isolated SAVR in patients with a diagnosis of aortic stenosis. All procedures between 2008 and 2020 are included.

Temporal trends of TAVI and SAVR procedures are illustrated in *Figure 1*. The overall rate rapidly increased for TAVI, while the rate for SAVR remained fairly constant.

**Figure 2:**
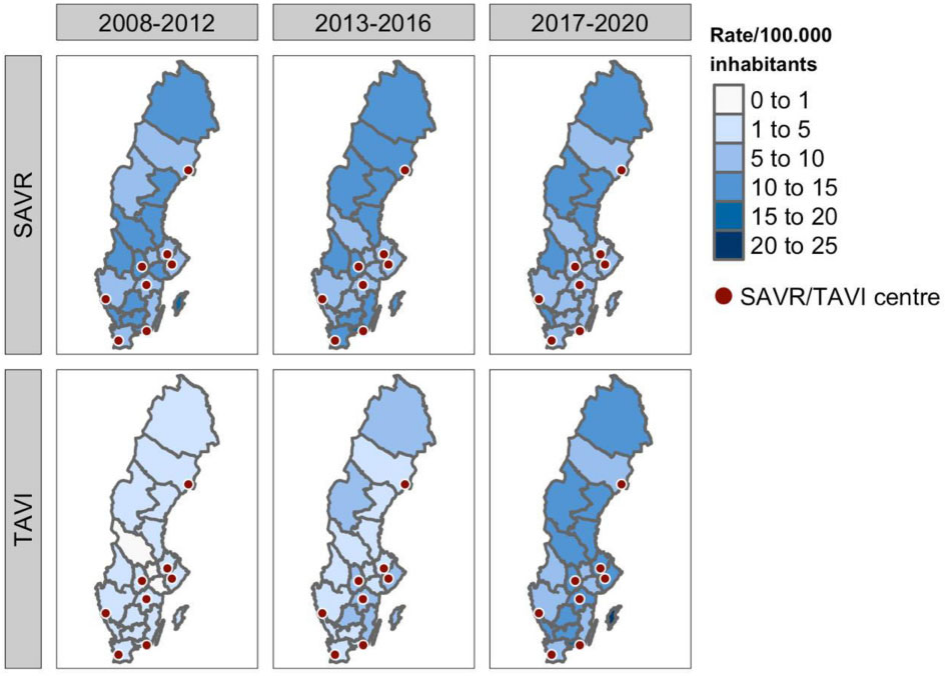
Temporal trends of TAVI and SAVR per 100 000 inhabitants, grouped by county of residence.

**Figure 3:**
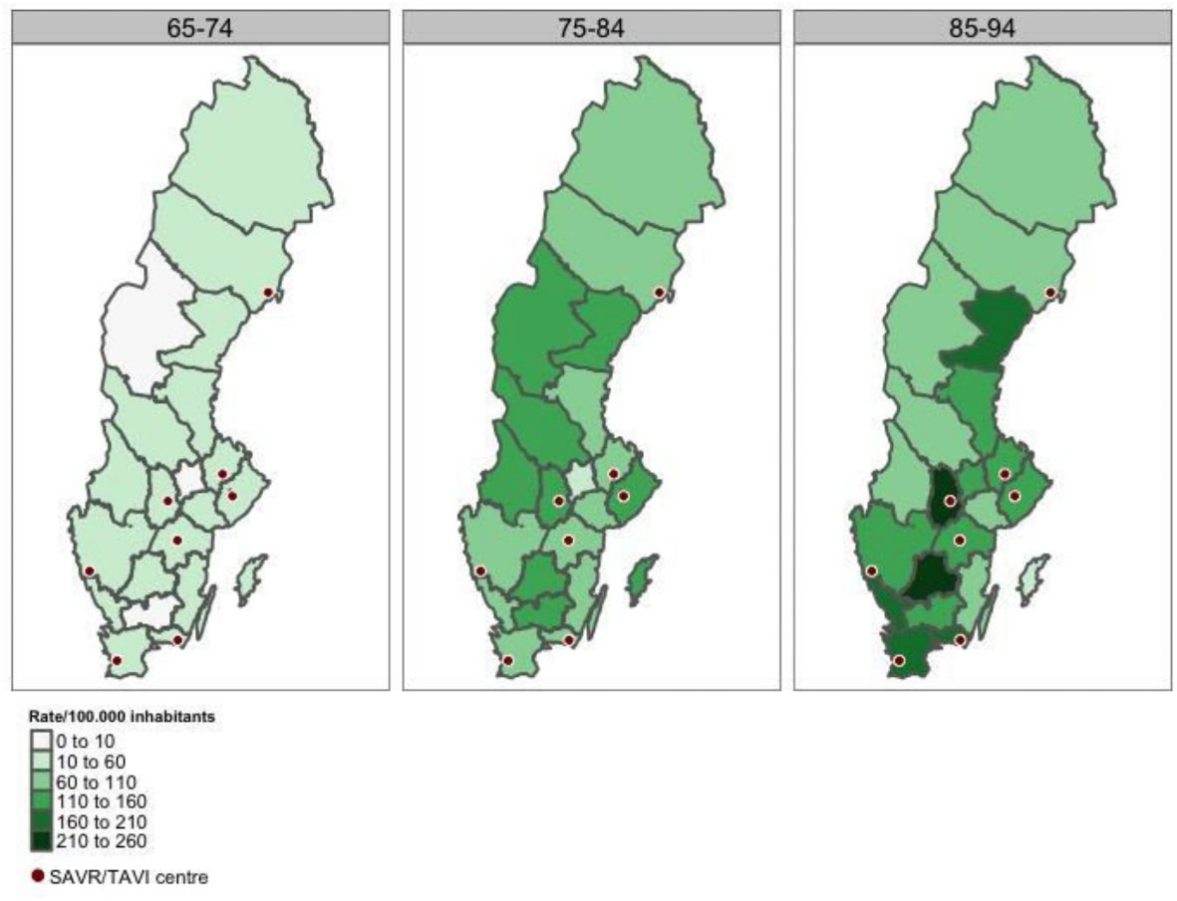
TAVI performed in 2020 per 100 000 inhabitants, grouped by county of residence and stratified by age groups.

As presented in *Figure 2*, regional rates for TAVI procedures per 100 000 inhabitants were not concentrated around TAVI centres. The Monte Carlo simulation of the Moran I statistic indicated no spatial correlation of the rates (*P* = 0.99). Standardized rates by age and sex were calculated (see Supplementary Appendix) using both direct and indirect methods with similar results. There was no statistically significant difference between counties with a local TAVI centre and counties without (Wilcoxon rank sum test, *P* = 0.74). As a sensitivity analysis, the analyses were repeated for patients with a procedure during 2020 and stratified into three age groups between 65–74, 75–84, and 85–94, respectively (see *Figure 3*). The results indicated no spatial correlation of the rates (*P* = 0.4, 0.46, 0.62, respectively).

For TAVI, the crude 30-day survival rate for the time period from 2008 to 2012 was 93.3% (95% confidence interval [CI] 91.7–95.0), while the rate was 98.1% (CI 97.8–98.5) for the time period from 2017 to 2020. For SAVR, crude 30-day survival rates in the same time periods were 98.1% (CI 97.8–98.5) and 98.7% (CI 98.3–99.1), respectively. Rates for all time periods are presented in *Table 2*.

**Table 2:** Thirty-day mortality rates and survival rates

	N	Person years	Events	Event rate/100 pyears	95% CI	Crude 30 day survival	95% CI
SAVR							
2008–2012	4536	368.07	84	22.82	18.2–28.25	0.981	0.978–0.985
2013–2016	3868	314.41	64	20.36	15.68–25.99	0.983	0.979–0.987
2017–2020	3412	277.97	44	15.83	11.5–21.25	0.987	0.983–0.991
TAVI							
2008–2012	842	65.81	55	83.57	62.96–108.78	0.933	0.917–0.950
2013–2016	1954	156.02	78	50	39.52–62.40	0.959	0.950–0.967
2017–2020	4472	362.13	86	23.75	19–29.33	0.981	0.977–0.985

Both the log-rank test testing county and a likelihood ratio test including an interaction term between county and year of procedure did not indicate heterogeneity in 30-day mortality.

**Figure 4:**
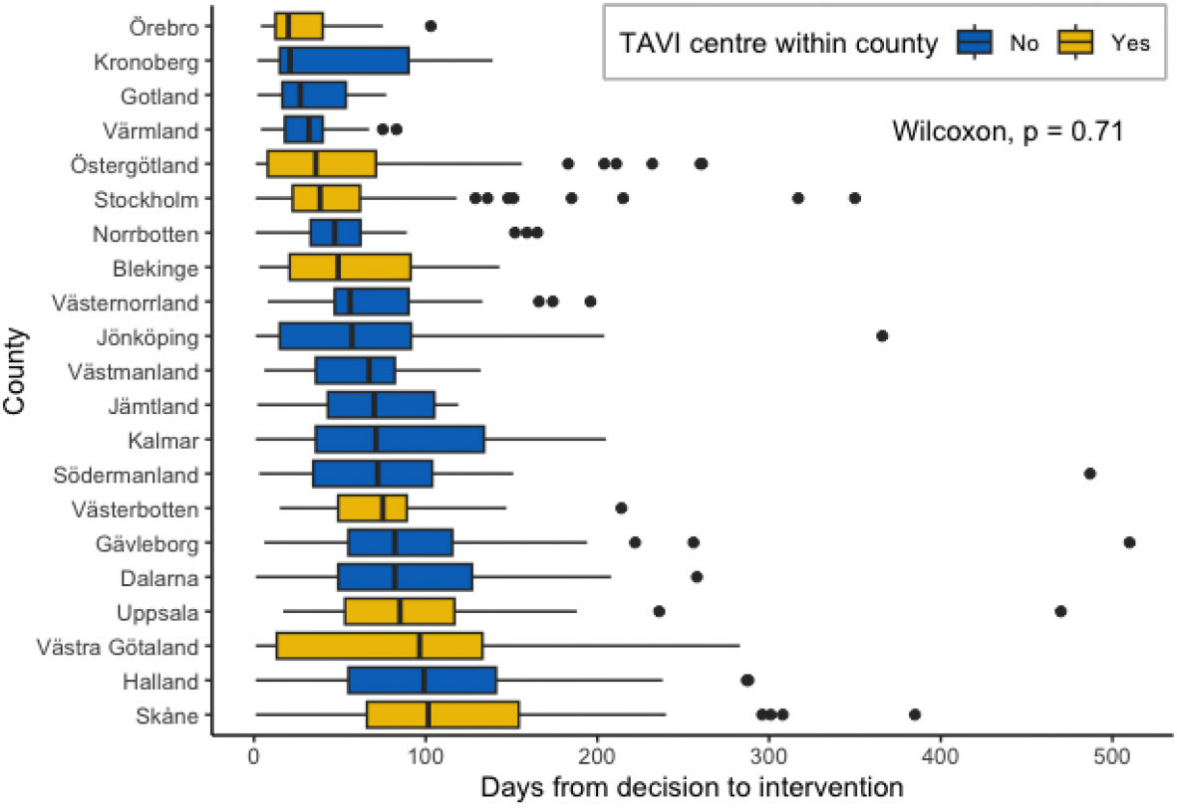
Time from decision to intervention for TAVI grouped on if county with or without TAVI centre. The box represents range, interquartile range, and median values.

The time from the decision to intervention for TAVI procedures performed in 2020 is presented according to county with or without TAVI centre in *Figure 4*. The overall mean and median waiting times were 67 days and 53 days, respectively. The median waiting times for regions with and without a TAVI centre were 61 and 58 days, respectively, without a statistically significant difference between counties with a local TAVI centre and counties without (Wilcoxon Mann‒Whitney test, *P* = 0.71). Excluding extreme outliers, possibly caused by erroneous data entry, did not alter the results.

## Discussion

There were no major geographical differences in TAVI availability or short-term mortality after TAVI. Throughout all counties, the number of TAVI procedures increased, and mortality rates decreased over time.

An association between a higher number of procedures and better outcomes has been suggested not only for TAVI but also for several medical conditions.^9,21,22^

Most countries have health care organizations structured in several levels. Interventions that are complex or rare are often concentrated in specialized centres, and this is well in line with the assumption that more specialized centres are expected to have more experience and better results of complex procedures; in other words, it is believed to be best for the patients. All resources for complication management should be available on site, e.g. in a TAVI context, thoracic surgeons. Nonetheless, this introduces a case mix where more severe cases are referred to specialized centres, thus making the comparison of actual outcomes difficult.^23^ However, in addition to medical considerations, there may also be economic reasons for the way health care is structured, as it might not be possible to have the same resources located everywhere.

Nevertheless, a possible concern with the concentration of resources is that this could lead to inequity, as people living far away have a higher threshold to become candidates for treatment. This is a consequence of the distance itself; patients could be more unwilling to undergo long transportation, although organizational aspects and patient characteristics influence to what extent.^10^

The findings in this study, where no regional differences in TAVI availability were indicated, are interesting in this context. Although all Swedish citizens have universal availability to tax financed, high-quality medical care,^24^ Sweden is a geographically large country, and in a European context, it is sparsely populated.^16^ Moreover, the Swedish health care organization is to a high extent decentralized. Most health care is managed by politically elected regional authorities, called county councils, who are autonomous regarding health care organization and tax management.^11,24^ In theory, this system could create a basis for regional differences and difficulties regarding the coordination of resources. One of the lessons from the COVID-19 outbreak was that the health care organization made it difficult to coordinate interventions.^25,26^

Nonetheless, in this study, no regional differences in availability to care or short-term mortality were found, which suggests that despite a highly decentralized health care system with local economic frameworks, a few centralized centres are sufficient to maintain an equal availability to TAVI.

Another possible concern with a limited number of centres is waiting times. Earlier studies have indicated mortality rates of 2% to 4% while on the waiting list for TAVI, although numbers as high as 14% have been reported.^9,27,28^ In this study, the mean and median waiting times were approximately 2 months without a significant difference between regions with or without a TAVI centre. However, the patients are registered in SWENTRY at the time of the procedure, and thus, patients who do not reach intervention are not captured by the registry. A better understanding of the consequences of the current waiting times in terms of mortality would require the identification of untreated patients with severe aortic stenosis eligible for TAVI, which was not possible with our available registry data. Consequently, while there was no difference in waiting times between TAVI centre regions and non-TAVI centre regions, further analysis of waiting times is not possible to perform with our data. Moreover, as the numbers of TAVI procedures are expected to keep increasing over time, the conditions may change, and capacity might become more of an issue. In fact, a waiting time of 2 months is already too long and needs to be shortened. However, in the SWENTRY annual report^29^ waiting times have remained fairly constant since 2018 despite a considerable increased number of procedures. This indicates that the existing TAVI centres have managed to increase their capacity and that other factors than the number of centres could be important to shorten waiting times. In order to be able to treat increasing numbers of patients, the existing centres need to continue to streamline their processes.

For PCI, a large-scale regionalization has been carried out in Sweden and other countries. A previously published meta-analysis of 23 studies reported no differences in outcomes between centres with or without on-site cardiac surgery.^30^ In our study, mortality and both interventional complications and complications during hospitalization decreased over time. In addition, in the publicly available yearly reports from SWENTRY,^29^ almost all major complications after TAVI, such as new permanent pacemaker, stroke, or bleeding events, are decreasing gradually. Most of these complications could also be treated without thoracic surgical expertise on site. One could therefore argue that a similar decentralization of TAVI comes with low risks in terms of complications and potential benefits in terms of availability. However, unlike PCI, TAVI procedures are almost exclusively elective and consequently not as time sensitive as acute medical conditions. Moreover, PCIs are performed in much greater numbers and do not face the same risk of dilution of competence.^31^

In 2015, a Swedish government official report was published with the (freely translated) title ‘Practice makes perfect’.^32^ The report advocates for a more centralized organization of highly specialized care. While the current ESC guidelines on valvular heart disease list the minimum number of procedures necessary to maintain good quality as an area where further research is needed, the report suggests a benchmark of at least 50 to 100 annual procedures per centre and at least 30 procedures per operator.^8,32^ Some of the Swedish centres are in the lower part of this interval,^29^ and this, together with the results of this study with similar availability and outcomes throughout the country, indicates no need for further decentralization as of today. If new centres are considered, this should primarily be in response to a higher demand where existing centres cannot increase their capacity.

## Limitations

Our study has several limitations. While adjustments for regional variations in age and sex were analysed, there could still be considerable differences in case mix between regions. Moreover, although the rates per 100 000 inhabitants were not concentrated in regions with TAVI centres, the study did not examine whether there were regional differences in the timing of intervention. That is, at what point in the disease progression were patients referred for intervention. In addition, the study analysed data grouped by county and did not include any variations in availability to TAVI within each county. Furthermore, as the number of counties was quite small, statistical tests should be interpreted with care.

## Conclusion

In this nationwide study, there was no evidence of regional heterogeneity in the availability of TAVI or mortality within 30 days of the procedure. The current system, with universal availability to health care and few specialized centres, seems adequate in providing equitable care for patients with severe aortic stenosis. Our results, if corroborated by further studies performed in different countries, could be helpful for the decision process on how to expand the TAVI volumes by preserving patient safety. A note of caution should be required before extending the TAVI procedures from tertiary to secondary health care centres.

## Supplementary Material

qcad076_Supplemental_File

## Data Availability

The data underlying this article cannot be shared due to ethical reasons.
