# Supplementary material for: Regional assessment of availability for transcatheter aortic valve implantation in Sweden: a long-term observational study
Source: Eur Heart J Qual Care Clin Outcomes. 2023 Dec 29;10(7):641–9. doi: 10.1093/ehjqcco/qcad076 (PMC11537233; doi:10.1093/ehjqcco/qcad076)
Supplement: qcad076_Supplemental_File [file qcad076_supplemental_file.docx]

**Supplementary appendix**

**Supplementary Table 1: Surgical aortic valve replacement baseline characteristics**

|  | **2008-2012** | **2013-2016** | **2018-2020** |
| --- | --- | --- | --- |
| n | 4052 | 4250 | 4632 |
| Age: n (SD) | 68.35 (12.16) | 68.49 (11.83) | 66.37 (11.81) |
| Sex: Female n (%) | 1642 (40.5) | 1694 (39.9) | 1615 (34.9) |
| Body Mass Index (kg/m2): (mean (SD)) | 27.09 (4.72) | 27.64 (6.71) | 27.75 (6.08) |
| Stroke: n (%) | 153 (9.0) | 323 (8.0) | 338 (7.5) |
| Diabetes: n (%) | 529 (14.4) | 699 (16.7) | 717 (15.6) |
| Hypertension: n (%) | 80 (59.3) | 30 (53.6) | 2455 (62.5) |
| Smoking: n (%) | 116 (35.3) | 710 (37.9) | 1579 (40.9) |
| Atrial fibrillation: n (%) |  |  |  |
| No | 316 (84.7) | 1793 (84.1) | 3733 (85.6) |
| Paroxysmal | 22 (5.9) | 149 (7.0) | 332 (7.6) |
| Persistent | 35 (9.4) | 191 (9.0) | 294 (6.7) |
| COPD: n (%) | 345 (8.9) | 361 (8.8) | 335 (7.3) |
| Pulmonary hypertension (mmHg): n (%) |  |  |  |
| <=30 | 806 (92.4) | 2902 (79.3) | 3585 (82.4) |
| 31-55 | 41 (4.7) | 602 (16.4) | 629 (14.5) |
| >55 | 25 (2.9) | 157 (4.3) | 136 (3.1) |
| PVD: n (%) | 274 (7.0) | 265 (6.4) | 200 (4.4) |
| Neuromuscular dis.: n (%) | 100 (2.6) | 151 (3.7) | 103 (2.3) |
| CABG: n (%) | 397 (10.2) | 343 (8.3) | 317 (6.9) |
| Left ventricular ejection fraction: n (%) |  |  |  |
| >50 | 1081 (75.6) | 3164 (78.0) | 3630 (80.5) |
| 31-50 | 286 (20.0) | 712 (17.6) | 711 (15.8) |
| 21-30 | 60 (4.2) | 133 (3.3) | 122 (2.7) |
| <20 | 2 (0.1) | 45 (1.1) | 46 (1.0) |
| Recent myocardial infarction: n (%) | 160 (4.1) | 142 (3.4) | 107 (2.3) |
| NYHA class: n (%) |  |  |  |
| I/II | 389 (41.2) | 1994 (52.2) | 2622 (58.5) |
| III | 488 (51.6) | 1667 (43.7) | 1679 (37.5) |
| IV | 68 (7.2) | 157 (4.1) | 178 (4.0) |
| eGFR (ml/min/1.73m2): mean (SD) | 74.82 (20.50) | 74.75 (19.75) | 77.58 (18.76) |
| Dialysis: n (%) | 36 (1.1) | 63 (1.5) | 51 (1.1) |

CABG= coronary artery bypass graft. PCI= Percutaneous coronary intervention. COPD=chronic pulmonary disease. PVD=peripheral vascular disease. eGFR=estimated glomerular filtration rate according to the CKD-EPI formula. NYHA class= New York Heart Association class. LVEF= left ventricular ejection fraction.

**Supplementary Table 2:**

|  |  | Direct standardization | | | glm standardization | | | |
| --- | --- | --- | --- | --- | --- | --- | --- | --- |
| **County** | **Observed rate** | **Exp rate n** | **Conf low** | **Conf high** | **Exp rate** | **Conf low** | **Conf high** | **Exp rate n** |
| **01** | 69,0270 | 71,1902 | 71,1901 | 71,1903 | 1,21 | 1,06 | 1,38 | 83,4227 |
| **03** | 54,1395 | 55,6373 | 55,6372 | 55,6375 | 0,94 | 0,68 | 1,29 | 50,7027 |
| **04** | 42,5752 | 42,0963 | 42,0961 | 42,0965 | 0,71 | 0,49 | 1,04 | 30,4340 |
| **05** | 61,1759 | 60,4871 | 60,4869 | 60,4872 | 1,01 | 0,78 | 1,32 | 61,9579 |
| **06** | 80,2525 | 78,3433 | 78,3431 | 78,3435 | 1,32 | 1,02 | 1,71 | 105,6674 |
| **07** | 60,3413 | 58,4349 | 58,4346 | 58,4351 | 0,98 | 0,66 | 1,45 | 59,0712 |
| **08** | 46,4823 | 45,4965 | 45,4963 | 45,4967 | 0,76 | 0,52 | 1,11 | 35,3963 |
| **09** | 72,0744 | 71,9693 | 71,9688 | 71,9697 | 1,22 | 0,67 | 2,21 | 87,8675 |
| **10** | 70,3197 | 66,8792 | 66,8789 | 66,8795 | 1,12 | 0,76 | 1,64 | 78,5140 |
| **12** | 64,0088 | 63,6719 | 63,6718 | 63,6720 | 1,07 | 0,91 | 1,25 | 68,3852 |
| **13** | 58,6625 | 57,5343 | 57,5341 | 57,5345 | 0,97 | 0,71 | 1,31 | 56,7245 |
| **14** | 46,1800 | 46,1610 | 46,1610 | 46,1611 | 0,78 | 0,66 | 0,92 | 35,8728 |
| **17** | 59,0990 | 58,1720 | 58,1719 | 58,1722 | 0,97 | 0,71 | 1,33 | 57,2607 |
| **18** | 73,9249 | 74,8480 | 74,8478 | 74,8482 | 1,25 | 0,93 | 1,67 | 92,2714 |
| **19** | 39,4227 | 37,9494 | 37,9492 | 37,9495 | 0,64 | 0,43 | 0,97 | 25,3457 |
| **20** | 60,5379 | 60,7391 | 60,7389 | 60,7392 | 1,01 | 0,75 | 1,38 | 61,3494 |
| **21** | 60,3536 | 59,9381 | 59,9380 | 59,9383 | 1,01 | 0,74 | 1,37 | 60,7960 |
| **22** | 82,5522 | 80,8905 | 80,8903 | 80,8907 | 1,36 | 1,02 | 1,81 | 112,1612 |
| **23** | 53,0504 | 53,4755 | 53,4753 | 53,4758 | 0,90 | 0,55 | 1,47 | 47,6449 |
| **24** | 50,9989 | 50,9258 | 50,9256 | 50,9260 | 0,85 | 0,59 | 1,23 | 43,5977 |
| **25** | 48,7493 | 48,2492 | 48,2491 | 48,2494 | 0,80 | 0,56 | 1,16 | 39,1837 |

The results of the standardized rates adjusted for age and sex are presented in **Supplementary Table 2**. For the direct standardization, no major differences were observed. When the histograms of the populations of the age and sex distributions in each county were examined, the results were relatively similar, thus supporting the result of the direct standardization.

For the Poisson regression model, the differences were larger. However, the confidence intervals were wide, and it is plausible that the model is inadequate, as the intervention ratio in the population is low. In the table, the numbers for median age (81 years) men are presented.

In summary, differences in the background population within each county did not crucially affect the results, and we chose to present crude analyses.

**Supplementary Figure 1:**


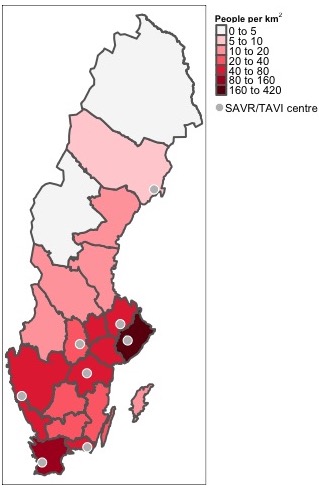


Population density in Sweden as of year 2020 as people per km^2^ grouped by region. The overall population density for the whole country was 25.5 people per km^2^.
